# Digital health for emotional and self-management support of caregivers of children receiving growth hormone treatment: a feasibility study protocol

**DOI:** 10.1186/s12911-022-01935-1

**Published:** 2022-08-13

**Authors:** Sergio Cervera-Torres, Francisco José Núñez-Benjumea, Antonio de Arriba Muñoz, Irene Alice Chicchi Giglioli, Luis Fernández-Luque

**Affiliations:** 1 Adhera Health, Inc, Palo Alto, CA USA; 2 Endocrinology Pediatric Unit, Hospital Universitario Miguel Servet, Zaragoza, Spain

**Keywords:** mHealth, Emotional wellbeing, Caregiver, Growth hormone, Pediatric endocrinology, Family-centered approach

## Abstract

**Background:**

Caregivers of children undergoing growth hormone treatment often face stress and stigma. In this regard, family-centered approaches are increasingly considered, wherein caregivers’ mental wellbeing is taken into account to optimize children’s health-related outcomes and behaviors (e.g., treatment adherence). Here, mindfulness and parenting-based programs have been developed to support the mental wellbeing of caregivers and, in turn, promote richer interactions with the children. Nevertheless, this type of program can face drawbacks, such as the scheduling and availability of family members. Recent digital health (DH) solutions (e.g., mobile apps) are showing promising advantages as self-management support tools for improving wellbeing and behaviors related to the treatments. Although, further evidence is necessary in the field of Growth Hormone Treatment (GHt). Accordingly, this study aims to examine the usability of a mobile DH solution and the feasibility of a DH intervention designed to promote emotional and mental wellbeing of caregivers of children undergoing GHt.

**Methods:**

This is a prospective mixed-methods (qualitative-quantitative) exploratory study composed of two sub-studies, including caregivers of children undergoing GHt. Sub-study one (SS1; n = 10) focuses on the usability of the DH solution (detecting potential barriers and facilitators) and an ad hoc semi-structured interview will be administered to the caregivers after using the DH solution for one month. Sub-study two (SS2; n = 55) aims to evaluate the feasibility of the DH intervention on caregivers’ perceived distress, positive affectivity, mental wellbeing, self-efficacy, together with the children’s quality of life and treatment adherence. All these parameters will be assessed via quantitative methods before and after 3-months of the DH intervention. Usability and engagement will also be assessed during and at the end of the study.

**Results:**

It is expected that significant amounts of data will be captured with regards of the feasibility of the DH solution.

**Discussion:**

The manuscript provides a complete protocol for a study that will include qualitative and quantitative information about, on one hand, the user-friendliness of the DH solution, and on the other, the effects on caregivers’ emotional, as well as, behavioral parameters in terms of the usability and engagement to the DH solution. The findings will contribute to the evidence planning process for the future adoption of digital health solutions for caregiver support and better health-related outcomes.

*Trial registration* ClinicalTrials.gov, ID: NCT04812665.

**Supplementary Information:**

The online version contains supplementary material available at 10.1186/s12911-022-01935-1.

## Background

Growth hormone is prescribed in children for a range of health conditions related to growth problems and short stature, and in particular patients with growth hormone deficiency compared with peers of the same age and sex [1, 2]. Furthermore, short stature in children has also been reported to be associated with psychosocial stress, social isolation, bullying, and impaired social competencies [3–5]. This problem extends to caregivers (usually parents), that have to deal with various stressors about child’s health, such as daily stressors (managing daily routine as ensuring treatment adherence) and emotional stressors (e.g., anxiety, worrying) [6, 7] and as well as social stigma [8, 9], ultimately caregivers can be affected by emotional fatigue and burnt out.Therefore, caregivers are at risk for developing psychosocial problems, such as anxiety and depression, which can reasonably lead to greater difficulties in handling effectively the child's illness and development. A child’ mental health development depends on their parents or caregivers who serve as primary sources of support in reaching an adaptive development. Studies on the importance of parents’ mental wellbeing in the child development have showed that a poorer parents’ mental wellbeing is related to a higher mental, emotional or development disability [10]. In particular, parental stress has been associated with less effective parenting [11] and poorer adherence of children to medical treatment [12]. On contrary, positive affect states have been found to be associated with health-related quality of life (HrQoL) in adolescents with Type I diabetes [13]. In a similar vein, self-efficacy as perceived control and capability to implement a course of action [14] and mental wellbeing have also been found positively related to pediatric health outcomes [15]. However, these factors has been extensively investigated within pediatric areas such as Type-I diabetes, cancer [16], and chronic pain [17], other pediatric areas such as growth conditions are misrepresented.

These are leading to serious consideration of family-centered approaches targeting the mental wellbeing of caregivers as a pivotal factor to optimize children’s health outcomes, such as treatment adherence [18–20]. Accordingly, cognitive behavioral therapy (CBT) and mindfulness-based cognitive therapy (MBCT) have demonstrated positive outcomes regarding stress and healthy quality of life of caregivers and children [21]. On one side, CBT includes the awareness of useless and negative thoughts and related-behaviours, reframing them with positive and useful thoughts and behaviours. On the other hand, MBCT aims to support patients to pay attention to their thoughts without judgment, accepting them and reducing the impact of negative and useless thoughts and behaviours, thus, providing tools to cope with the stressful situations [22, 23]. In this theoretical context, psychoeducational modules, as well as activities such as mindfulness, have been used, showing a decrease of stress and an increase in mental wellbeing. According to the previous evidence, mindfulness activities specifically designed to improve parents' self-efficacy and stress management have been shown to promote richer interactions with the children [21, 24]. In a similar vein, standardized parenting programs have been developed to promote positive parenting skills (e.g., problem-solving and communication techniques) [25].

However, despite these positive results, parents often experience practical barriers for participation in this type of interventions, such as travelling distance, and timing costs, looking for resources to take care of the children (e.g., babysitters), and absence from work [26, 27].

To overcome these issues, digital health (DH) solutions are enabling more accessible self-management supports and less costly family-centered intervention modalities [28–30]. A few recent pilot and feasibility studies are showing positive outcomes on the efficacy of caregiver interventions of children with chronic conditions, such as diabetes, and pediatric transplants through mobile apps [28, 29]. Similarly, other studies using web applications are showing that DH interventions are feasible to deliver training programs targeting, among others, parenting stress, problem-solving skills, protective parenting behavior, or self-efficacy of caregivers of children with chronic conditions (e.g., cancer, or obesity; see the review [31]). Interestingly, to our knowledge, none of the studies delivered a mobile-based DH intervention, via smartphone for caregivers of children undergoing GHt. Starting from these premises, the aim of this study is to investigate the usability of the mobile DH solution, called Adhera® Caring, and the trial design and feasibility of Adhera Caring intervention evidence-based on CBT and MBCT for caregivers’ mental wellbeing. Accordingly, the study protocol targets to obtain qualitative and quantitative answers to the following three research questions and derived hypotheses:

1. What is the overall usability experience of caregivers using Adhera Caring?
  Hypothesis 1: Participants will show an acceptable usability experience of the DH solution.
2. What effect does the intervention through Adhera Caring have on the levels of psychological distress, mental well-being, self-efficacy, of caregivers of children undergoing GHt for short stature?
  Hypothesis 2: The intervention will improve the levels of psychological distress, and will promote positive affect, self-efficacy, and mental wellbeing.
3. Which insights can be obtained regarding parental concerns, the children’s quality of life, and adherence to treatment?

Hypothesis 3: It can be expected that promoting levels of positive affect, self-efficacy, and mental wellbeing will be positively associated with children’s quality of life and treatment adherence.

### The Adhera® Caring digital health intervention

Own prior research has built upon mental health and technology acceptance theoretical frameworks [32] to test behavioral change and mental health support via digital techniques within health-related areas such as smoking cessation [33]. We developed a digital solution incorporating three main components. An educational health content component provides general health information about a particular condition and information on self-care and stress management. The solution also incorporates a mental-wellbeing module, which also includes psychoeducational information and different actionable activities such as attention exercises based on mindfulness. Finally, a recommender system, based on artificial intelligence (AI), which delivers short messages in the form of quick actionable suggestions or educational reinforcement.

Building upon this structure, the Adhera Caring is a DH intervention mobile-app-based that has been specifically designed to support caregivers of children undergoing a GHt for short stature with regards of reducing psychological distress and improving their overall mental wellbeing, positive affect, and self-efficacy. Accordingly, the Adhera Caring DH intervention program includes: a) a psychoeducational module; b) a mental wellbeing module to reduce psychological distress and to promote positive affect and mental wellbeing; c) a behavioral change module aiming at providing brief psychoeducational messages, promoting healthier lifestyles and self-efficacy.

### Adhera Caring: psychoeducational modules

**Fig. 1:**
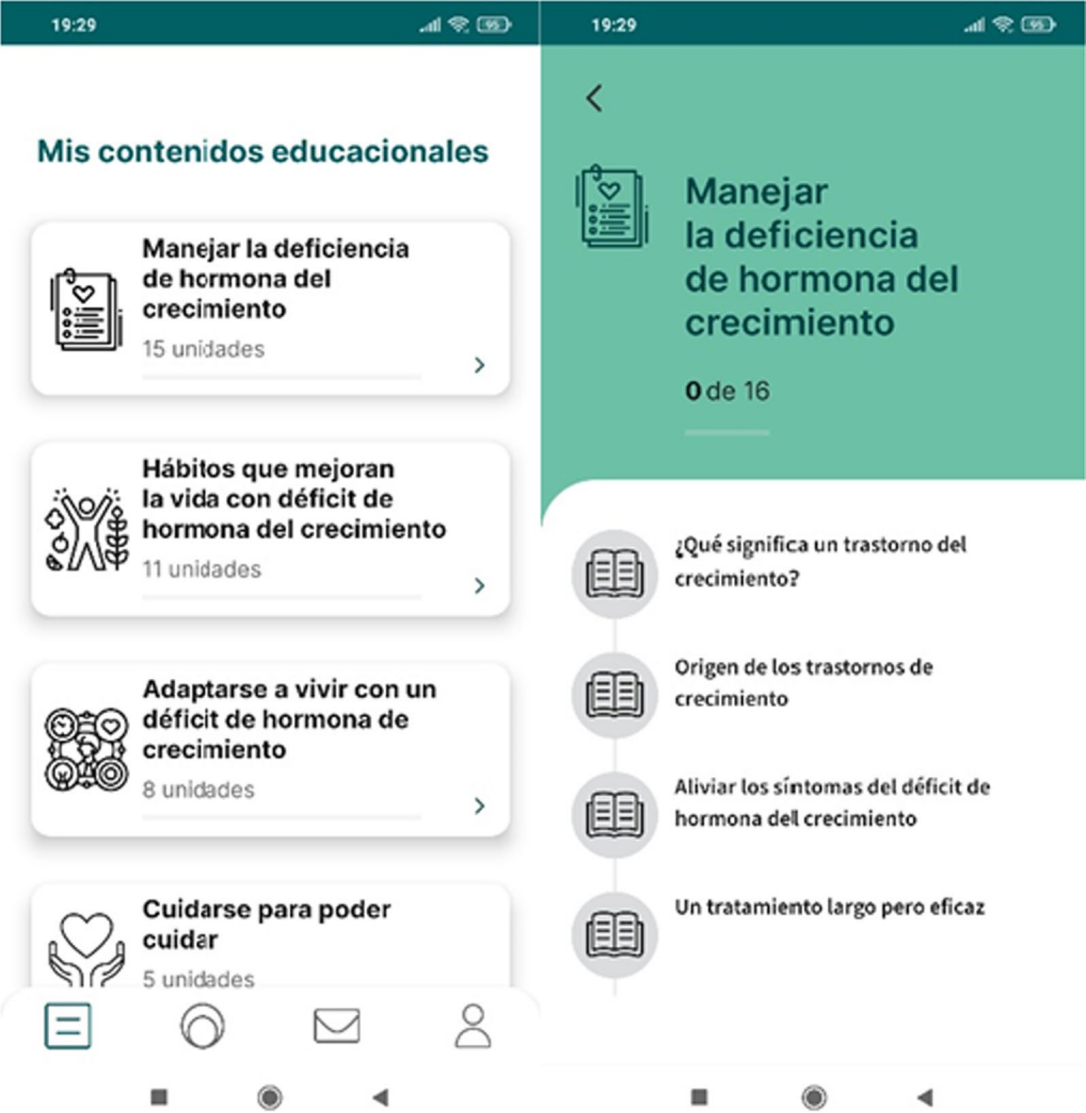
Adhera Caring: psychoeducational modules

**Fig. 2:**
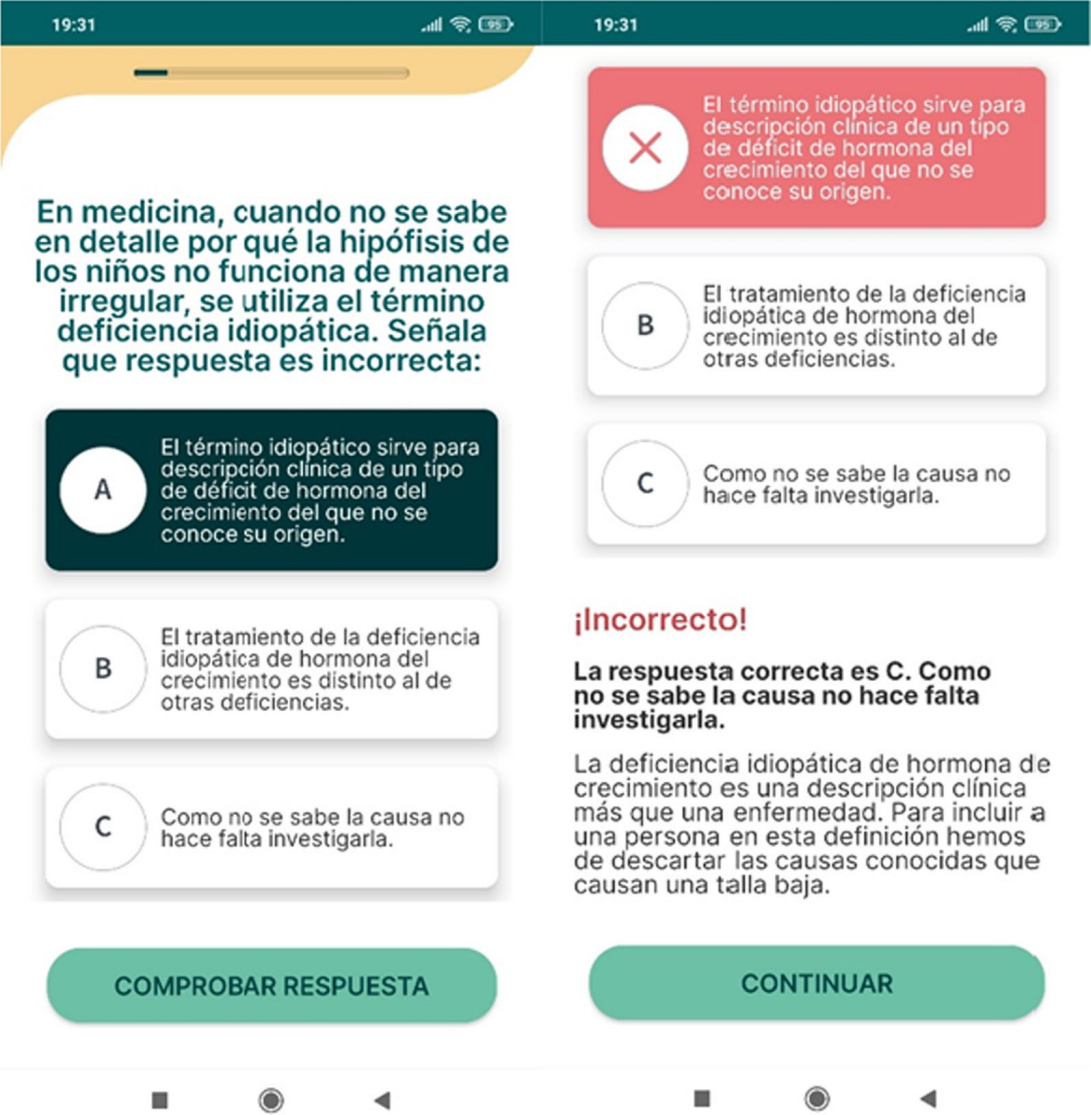
Adhera Caring: The quiz unit (gamification element) with the explanation of the answer

The psychoeducational modules have been designed to provide specific educational contents on childhood growth disorder and the related GHt, as well as benefits and secondary effects of the GHt, and problem-solving suggestions on how to support the children during the entire GHt process. This content includes 39 units divided into four sections) managing growth hormone deficiency; b) habits that improve life with growth hormone deficiency; c) adjusting to living with a growth hormone deficiency; d) taking care of yourself to be able to take care of your child (Fig. 1). The read contents will be marked with a tag and a progress bar appear according to the read contents. At the end of each module, the last unit consists of a quiz with multiple choices. After each answer, an explanation will be provided to the user (Fig. 2). All the material contents have been setup according to the Patient Education Materials Assessment Tool (PEMAT) guide on the understandability and actionability materials.

### Adhera Caring: the behavioral change module

**Fig. 3:**
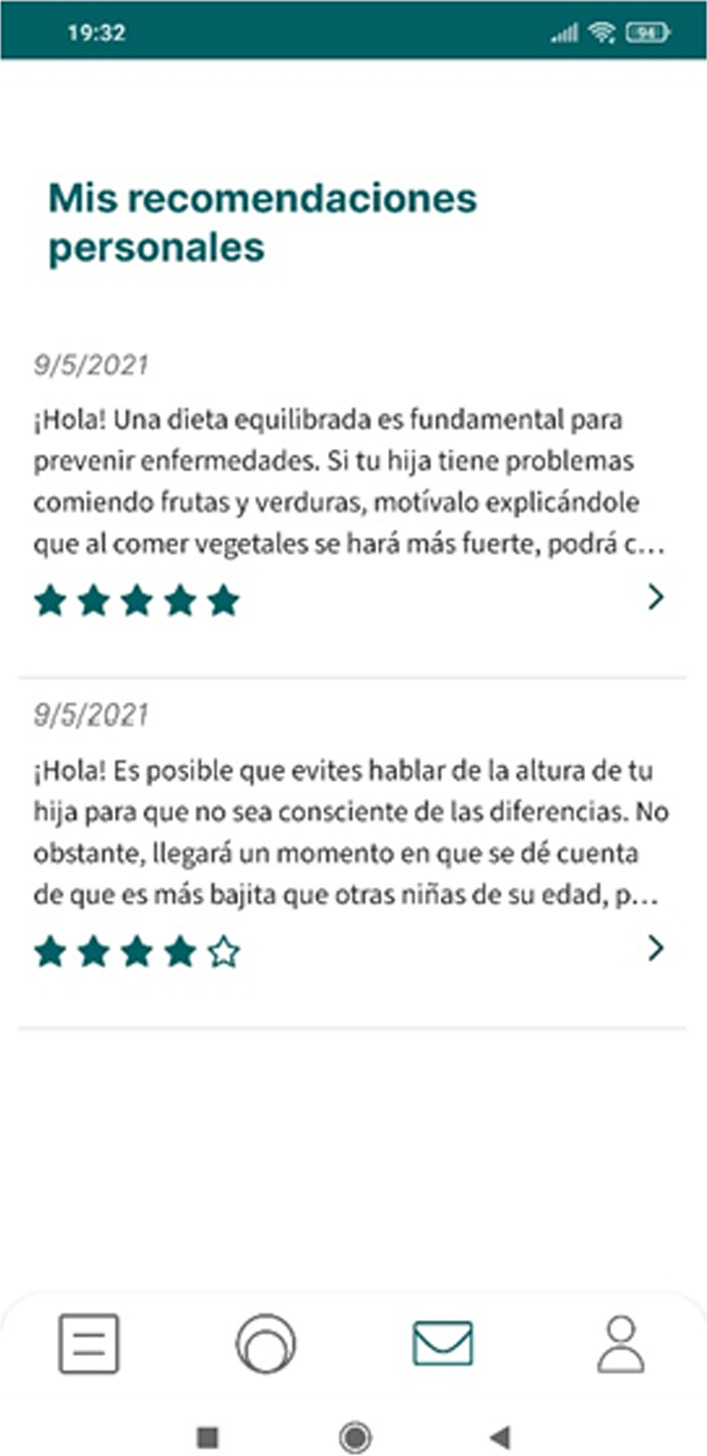
Adhera Caring: Motivational messages and ratings

The behavioral change module reinforces the psychoeducational module by delivering brief educational messages on the childhood growth disorder, as well as support in managing psychological distress, improving self-efficacy (caregiver perception of their ability to manage child-related stressful situations e.g., facing fear to the treatment), personal skills for self-regulation (in stressed and anxiety events). It also provides lifestyles suggestions and action planning (actions to perform the desired behaviors and coping or maintaining planning) that will be sent to the patient with a set frequency (one daily; Fig. 3). The messages have been defined and validated by clinicians and behavioral change experts. When a message will be sent by this module, patients can then read them from an inbox of messages in their Adhera Caring app. The sent messages will be selected by the artificial intelligence of the Adhera Precision Digital Companion™ Platform to maximize the interest of the message for each patient—so each patient will receive tailored messages. Each message can then be rated between 1 and 5 stars, as feedback for the system to learn about the patients' preferences and improve their future recommendations.

#### Adhera Caring: mental well-being module

**Fig. 4:**
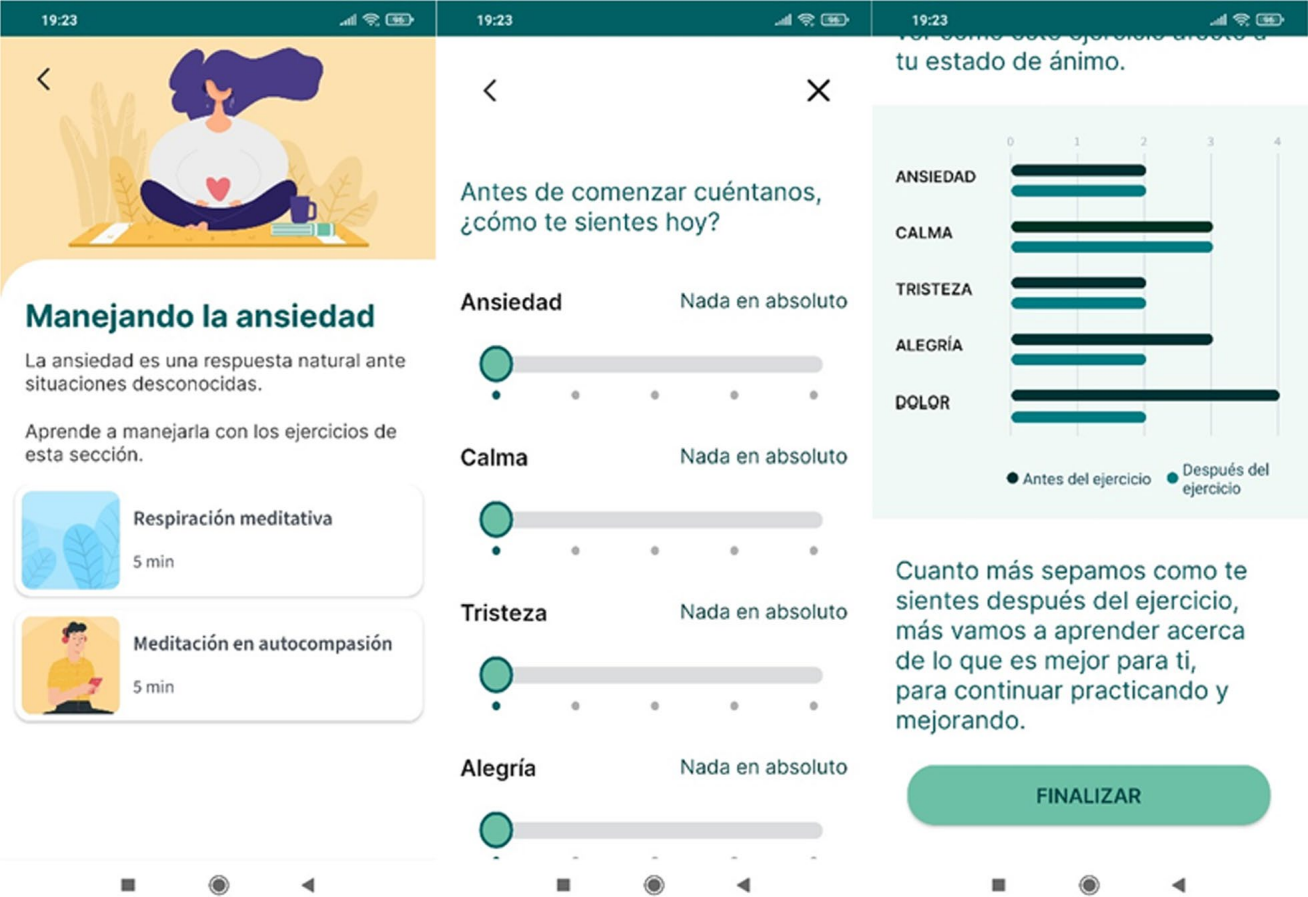
Mental Wellbeing activities and emotional pre-and post-evaluation

The mental wellbeing module provides patients with evidence-based CBT and MBCT-based activities to improve their mental wellbeing. They include visually guided activities to perform using the phone, audios where a psychologist instructs the patient how to relax, and audio files with relaxing music. In addition, other mental-wellbeing activities are also explained to be done without the need of using the smartphone. Pre- and post- each activity, user will rate on a 1–5-Likert scale mental wellbeing emotional states. A final report on the before and post mental wellbeing rates will be showed to the user (Fig. 4).

#### Digital health ecosystem for treatment adherence

The recruitment for Adhera Caring Digital Health Intervention relies on the existing digital health ecosystem around the growth hormone treatment with recombinant human GH (r-hGH, somatropin, Saizen®, the healthcare business of Merck KGaA, Darmstadt, Germany) [34], that includes the use of an automated electronic injection device called Easypod™, that can be used voluntarily by the patient (and/or caregiver) to administer the growth hormone therapy [35]. The device transmits the data (time, date and dose) to a secure web-based portal called Easypod Connect where clinicians can monitor and manage herence levels of patients. This platform will be used to screen potential participants in the study with sub-optimal levels of adherence, and will be used to capture information on the impact of the intervention in adherence.

The solution Adhera Caring is part of the Adhera® Health  Precision Digital Companion™ Platform, which has been developed using the best practices with regards of data protection and quality management in accordance with the guidelines of the ISO 27001 and ISO 13465.

## Methods

### Design and setting

This is a prospective mixed-methods usability and feasibility study intervention, see Additional File 1 for SPIRIT diagram. The study will be composed of two single group sub-studies (SS1 and SS2) targeting caregivers of children undergoing GHt in accordance with general clinical practice in Spain and the Miguel Servet Children’s University Hospital (Zaragoza, Spain) where patients are already supervised by a pediatric endocrinologist.

The goal of sub-study 1 (SS1; n = 10) is to gain qualitative insights about psychological burdens experienced by caregivers of children undergoing GHt, and barriers/facilitators after using the DH solution for one month. Insights from SS1 will serve to provide an optimized version of the mobile solution for its use in sub-study 2 (SS2; n = 55). In this case, psychological parameters in terms of psychological distress, positive affect, mental wellbeing, and self-efficacy will be targeted and assessed before and after using the DH solution (pre-post design). Children’s quality of life, treatment adherence, and system usability.

### Sample size

In qualitative research, the sample size cannot be conclusively estimated since it is determined by the saturation of the data acquired during the analysis of the qualitative data (e.g., semi-structured interviews). However, the sample size in SS1 has been estimated as n = 10, based on similar studies assessing the acceptance of a mobile solution, wherein the sample size for interviews and feasibility pilots was between 10–20 participants [36, 37]. Data saturation in the qualitative thematic analysis is expected to be achieved in less than ten subjects due to the focused nature of the study. In the case that this saturation is not achieved, the sample will be extended to include ten more participants.

In SS2, a sample size of n = 55, has been estimated by the GPower v.3.1.9.2 software. Specifically, a study design with 55 participants is appropriate to detect a low-to-medium effect size (Cohen’s *d* = 0.40) with a probability of α = 0.05 associated with a power of 1—ß = 0.08. The estimation is based on mean group differences between the baseline assessment and 3-months of follow-up (i.e., two-sided one-sample t-test).

Participants will be followed after Informed Consent Form (ICF) signature at the hospital to the end of the data collection period. It should be noted that in SS1, participants would engage with the solution for 1 month after recruitment. Since SS1 mainly focuses on usability, we estimated a period of one month, so that participants have a reasonable time to test the DH solution and subsequently rise concerns, barriers or facilitators when using the app. During this one-month observation time, participants will be supported if any doubt or concern arises.

In SS2 participants will be able to engage with the solution, for 3 months. The timing has been established on the standard mindfulness-based training programs that last a minimum of eight weeks [38]. Since the DH solution is self-administered, we added one extra month so that participants have more time to engage with the proposed activities. Major protocol amendments, if any, will be rigorously handled and updated at the ClinicalTrials.gov registry. The present study protocol has been elaborated following the recommendations for study protocols [39] based on the CONSORT extension statement for feasibility trials and the Standard Protocol Items: Recommendation for Interventional Trials (SPIRIT; see Additional file 1).

### Eligibility criteria

The sample of the study will be identified according to the following inclusion criteria: a) caregivers are legal guardians of children who receive GHt in accordance with approved indications in Spain, and b) adherence to GHt monitored in the last month prior to enrollment indicates a ratio less than 85%, since it has been considered as an index of relatively sub-optimal adherence [40, 41]. Also, participation in the study requires: c) explicit agreement on data sharing regarding adherence to GHt gathered through the Easypod Connect, d) participants must be able to interact and willing to install, the DH solution of the study in their smartphone, and e) participants must sign the specific ICF for the study.

Consequently, exclusion criteria define those candidates without an Android smartphone or not being able to interact with it cannot participate in the study, because the solution only works through this operative system. Moreover, only one legal guardian per child can participate in the study, and participants in SS1 will not take part in SS2.

### Recruitment

Participants will be recruited from the Pediatric Endocrinology unit at the Miguel Servet Children’s University Hospital. This public hospital provides pediatric health attention to the regional area of Zaragoza (Spain), which covers an approximate 367,110 inhabitants. Participants will be recruited at the pediatric unit by the study site investigator, which is a pediatric endocrinologist experienced in the management of children with growth disorders and their associated team. Enrollment in the study will be based on the information available in the Electronic Health Record (EHR) of potential participants. Whenever a candidate is found suitable, participation in the study will be offered during routine consultations. Participants will sign an informed consent during routine clinical visits to the center. For accessing treatment, adherence data (via Easypod-Connect) of children aged 12 years old and over, a specific ICF will need to be signed according to the Spanish regulation.

Participants will be free to discontinue the study at any time. Reasons to abandon the study will be recorded in the Electronic Case Report Form (eCRF; e.g., no perceived benefit of the mHealth solution, failure to attend the scheduled visit, etc.).

### Planned study DH intervention

SS1 will consists of three visits:

- Enrolment: participants will sign the ICF and complete a paper-based questionnaire on demographic variables. The estimated recruitment duration has been established in a time of 3 months.
- Baseline assessment: participants will complete a paper-based questionnaire on anxiety, depression and stress (for more details, see the 2.5 section on the outcome variables). After answering to the questionnaire, participants will download on their own smartphone the DH solution Adhera Caring and a training on how to use this tool will be provided. Participants will take Adhera Caring with them to home and could use it as often as they like.
- Post-assessment (follow-up visit): after one month, during the standard care visit, participant will participate in a semi-structured interview.

**Fig. 5:**
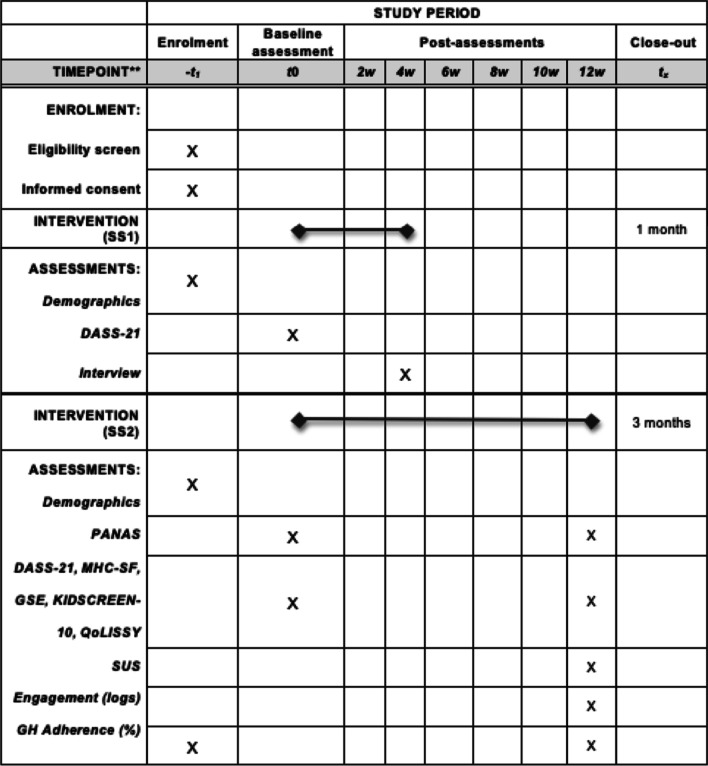
Standard Protocol Items: Recommendations for Interventional Trials (SPIRIT). Schedule of enrollment, interventions, and assessments planned in the study

SS2 will include the same visits of the SS1 and the estimated recruitment duration has been established in a time of 6 months. Psychological distress, positive affect, self-efficacy, mental wellbeing, and children’s quality of life will be collected at baseline and follow-up visits using paper-based questionnaires. In addition, a system usability questionnaire will also be administrated during the last visit (Fig. 5).

### Outcome variables

#### Research question 1 (Primary outcome)

Initially, the study aims to understand the psychological burdens experienced by caregivers of children undergoing GHt, and the perceived barriers/facilitators concerning the adoption of a DH solution. To do so, a group of ten caregivers will engage with the DH solution for 1 month. Subsequently, an ad hoc semi-structured interview based on mental health and technology acceptance theoretical framework [42] will serve to gather qualitative information about those user experiences (SS1; see Additional File 2). Such qualitative information will be contextualized by minimal quantitative data in terms of demographics (caregiver: age, gender, marital status, employment status, education level according to the European Qualification Framework [43]; children (patient): age, gender) screening of caregivers’ current level of psychological distress and usability and engagement with the solution. The following instruments to assess these outcomes:

- *Psychological Distress*: DASS-21 (Depression, Anxiety, Stress Scale; [44, 45]; Cronbach’s alpha 0.90—0.96). The scale contains 21 items with three subscales assessing symptoms of depression (e.g., I felt I wasn’t worth much as a person), anxiety (e.g., I felt scared without any good reason), and stress (e.g., I found it difficult to relax) during the last week. Scores range from 0 (never experienced) to 3 (almost always). Total scores represent general psychological distress.
- *System Usability*: SUS (System Usability Scale; [46]; Cronbach’s alpha 0.92). 10-item scale based on a 5-point Likert scale measuring strength and agreement of usability (e.g., I thought the solution was easy to use).
- *Engagement:* Measured by means of user logs including mobile-based user profiling.

#### Research question 2 (Primary and Secondary outcomes)

SS2 will assess, as a primary outcome, caregivers’ positive affect related to the use of the DH solution. In this case, caregivers will engage with the solution for three months.

- *Positive Affect*: the positive affect subscale of the PANAS (Positive and Negative Affect Schedule (PANAS; [47]; Cronbach’s alpha 0.92) will be used. This subscale is composed of 10 items and will evaluate experienced positive states (e.g., enthusiastic) from 1 (“never”) to 5 (“very much”) during the last month.

SS2 will also explore whether using the DH solution affect psychological distress by the DASS-21 and:

- *Mental Wellbeing*: the MHC-SF (Mental Health Continuum in Short Form; [48, 49]; Cronbach’s alpha 0.94) evaluates from 0 (“never”) to 5 (“Every day”) the felt experience during the last month regarding three dimensions of mental wellbeing: emotional (e.g., I felt happy), psychological (e.g., I liked most parts of my personality), and social wellbeing (e.g., I belonged to a community).
- *Self-Efficacy*: the GSE (General Self-Efficacy Scale; [50, 51]; Cronbach’s alpha 0.90) is a 10-item scale assessing perceived self-efficacy (e.g., “I can solve most problems if I invest the necessary effort”) from 1 (“not at all true”) to 4 (“exactly true”).

### Research question 3

Finally, SS2 will also explore several dimensions related to the children’s quality of life and GHt adherence.

- The parents' version of the *QoLISSY* (Quality of Life Inventory Short Stature Youth; [52]; Cronbach’s alpha 0.78—0.96) will be used to assess QoL dimensions. It includes three core sub-scales, physical (6 items; e.g., Because of my child's height (s)he has more trouble reaching things than others his/her age), social (8 items; e.g., Because of his/her height (s)he is treated differently), and emotional (8 items; e.g., Because of his/her height (s)he is shy) aspects of HrQoL. Additional sub-scales cover coping (10 items; e.g., When (s)he feels bad about his/her height (s)he tries to think of something nice), beliefs about height (4 items; e.g., My child believes that being tall helps in life), and specific questions for parents (11 items). These items refer to parents’ worries about the future of their child in relation to his short stature and effects of the child’s short stature on the parents’ feelings (e.g., My child worries about whether (s)he will grow enough). The items are responded from 0 (“not at all”) to 4 (“always”).
- *Children’s Quality of Life*: the parent version of the KIDSCREEN-10 ([53]; Cronbach’s alpha 0.82) contains 10 items (e.g., has your child felt well and fit?) in 5 point Likert response format, from “not at all” to “extremely”.

Finally, behavioral parameters in terms of usability (by the SUS) engagement (by logs) with the DH solution will be measured together with children’s adherence to the treatment through the Easypod Connect (time, date and dose) [35]

### Data management and quality control

All the data gathered in the study will be recorded in an electronic case report form (eCRF). This eCRF will be built upon the OpenClinica suite Community v3.4. The main purpose of the eCRF is to obtain data required by the study in a complete, accurate, legible, and timely manner. The data in the eCRF will be consistent with the relevant source documents.

The clinical investigator is responsible for ensuring that the data collected in the course of this study is accurate and documented appropriately on all applicable forms. Data will be processed, evaluated, and stored in anonymous form in accordance with the data protection regulation. The clinical investigator must ensure that the eCRFs and any other associated documents contain no mention of any subject names. The data will be entered into a validated database. Database lock will occur once quality control and quality assurance procedures and coding activities have been completed.

### Data analysis plan

Qualitative data in SS1 will be gathered by means of video/audio interview recordings, which need to be transcribed and coded, first, to be able to manage a more standard dataset. Subsequently, all recordings will be deleted to preserve participants’ anonymity. At least two different reviewers conduct data analyses, independently. Through the iterative process, themes and subthemes will be identified and coded as barriers or facilitators associated with the use of the eHealth solution (Deductive part). Thematic content analysis will be performed afterwards (Inductive part) (37, 54). Descriptions of demographic, clinical, and usability data will be reported in a specific section.

Quantitative data in SS2 will be gathered in a different dataset. Descriptions of demographic and the study outcomes (frequencies, mean, standard deviation, median, maximum-minimum values data, skewness, and kurtosis) will be reported in a specific section. To analyze intervention effects on the primary outcome (i.e., Positive Affect), one sample t-test between baseline and 3-month follow-up scores will be performed. Similarly, pre-post data regarding the secondary outcomes (I.e., psychological distress, self-efficacy, mental wellbeing, and children’s quality of life) will also be examined by t-tests. Bonferroni correction will be used to account for multiple comparison testing. A non-parametric approach (I.e. Mann- Whitney *U* test) will be used in the case that the data shows departures from normality distributions.

In addition, exploratory analyses will also include correlations to identify relevant associations between demographic, primary, secondary outcomes, as well as, childrens’ QoL, treatment adherence, and usability.

## Results

The recruitment process for the SS1 is planned to start by June 2021 and the qualitative analysis is expected to be done by September 2021. Regarding SS2, the recruitment process is planned to start by January 2022 and the primary impact analysis is foreseen to be conducted by February 2023. This protocol describes a novel approach to the mental wellbeing of caregiver of children undergoing a stressful treatment such as the GHt. Furthermore, the protocol presents a scalable tool showing how a collaboration between companies and public health care system might promote such technology into the global health care market.

According to our research questions and hypothesis, we will expect that:

- In SS1, the usability of the designed DH solution will be acceptable. Furthermore, in SS2 we expect high scores in the SUS (Hypothesis 1).
- A decrease on the levels of psychological depression, anxiety, and measured by the DASS-21 (Hypothesis 2)
- An improvement of positive affect (by the PANAS), self-efficacy (by the GSE), and mental wellbeing (by the MHC-SF), and particularly, emotional and psychological wellbeing. (Hypothesis 2)
- Finally, those psychological variables are positively associated with the children’s quality of life and treatment adherence. (Hypothesis 3).

## Discussion

The study protocol aims to investigate the usability user experience and the intervention feasibility of Adhera Caring DH that has been developed upon principles of technology acceptance [25]. The results of this study will provide relevant information to support the mental well-being of caregivers, in this case of children undergoing GHt but with the long-term goal that the Adhera Precision Digital Companion Platform will be used and its effectiveness evaluated by caregivers of children with different clinical conditions, such as diabetes mellitus and chronic diseases with prolonged subcutaneous treatments (e.g., immunodeficiencies, rheumatological diseases). CBT and MBCT have been proposed as the main techniques to increase, on one hand, the caregivers’ awareness of their children’s medical condition, their own useless and negative thoughts and related-behaviours, reframing them with positive and useful thoughts and behaviours; and on the other, to provide activities to support patients to pay attention to their thoughts without judgment, accepting them and reducing the impact of negative and useless thoughts and behaviours, thus, providing tools to cope with the stressful situations. Changes of caregivers’ negative thoughts and affect states into positives could have relevant implications into the treatment adherence, quality of life and children development [6, 15]. Indeed, caregivers represent the first source for a healthy children mental wellbeing development and in the current healthcare system it is hard to provide caregivers’ support and DH interventions can provide it on a larger scale which would be reflected in the future quality of life in the long term. However, more research is needed on both the usability and the effectiveness of this type of intervention to achieve a randomized control trial and Adhera Caring fits into this research framework.

According to this, it is also relevant to present some limitations of the actual DH research and the future challenges. Among these limitations, the intervention does not investigate potential effects of each of the three modules (I.e., educational, behavioral change, and mental wellbeing) in isolation but as a whole. In this regard, own prior research demonstrates that motivational messages following a behavioral-change model can be effective in the context of smoking cessation [26]. Therefore, further research is needed to clarify the concrete impact of these components in the particular context of a DH solution targeting caregivers of children undergoing GHt. Another limitation is that children involved in the study can be at different GHt intervention stages. In this regard, it is reasonable that caregivers’ concerns and mental health (e.g., psychological distress) could also be heterogeneous. Future studies could investigate effects of the intervention in recently diagnosed children so that parent can get presumably more profit of the content delivered by the DH solution.

## Supplementary Information

**Additional file 1.** SPIRIT Diagram explaining study design.

**Additional file 2.** Semi-structured qualitative interview designed for the qualitative sub-study 1.

## Data Availability

Not applicable.

## Abbreviations

AI: Artificial intelligence
CEICA: Aragonese ethical committee for clinical research
CBT: Cognitive beahavioral therapy
CONSORT: Consolidated standards of reporting trials
DASS: Depression, anxiety, stress scale
eCRF: Electronic case report form
EHR: Electronic health record
GDPR: General data protection regulation
GHt: Growth hormone therapy
GSE: General self-efficacy
HCP: Healthcare professional
HrQoL: Health-related quality of life
ICF: Informed consent form
ICH GCP: International conference on harmonization and good clinical practice
IRB: Institutional review board
LMM: Linear mixed model
MANOVA: Multivariate analysis of variance
MBCT: Mindfulness-based cognitive therapy
MHC-SF: Mental health continuum-short form
PANAS: Positive and negative affect scale
QoLISSY: Quality of life in short-statured youth
SGA: Small for gestational age
SPIRIT: Standard protocol items: recommendation for interventional trials
SS1: Sub-study 1
SS2: Sub-study 2

## References

[1] Brod M, Alolga SL, Beck JF, Wilkinson L, Højbjerre L, Rasmussen MH (2017). Understanding burden of illness for child growth hormone deficiency. Qual Life Res.

[2] Quitmann J, Behncke J, Dörr H-G, Willig RP, Wüsthof A, Stahnke N (2012). Gesundheitsbezogene Lebensqualität und psychische Gesundheit von kleinwüchsigen Kindern und Jugendlichen. Z Für Med Psychol.

[3] Bullinger M, Koltowska-Häggström M, Sandberg D, Chaplin J, Wollmann H, Noeker M (2009). Health-related quality of life of children and adolescents with growth hormone deficiency or idiopathic short stature–part 2: available results and future directions. Horm Res Paediatr..

[4] Visser-van Balen H, Sinnema G, Geenen R (2006). Growing up with idiopathic short stature: psychosocial development and hormone treatment; a critical review. Arch Dis Child.

[5] Voss LD, Mulligan J (2000). Bullying in school: are short pupils at risk? Questionnaire study in a cohort. Bmj.

[6] Silva N, Bullinger M, Sommer R, Rohenkohl A, Witt S, Quitmann J (2018). Children’s psychosocial functioning and parents’ quality of life in paediatric short stature: The mediating role of caregiving stress. Clin Psychol Psychother..

[7] Gérain P, Zech E (2018). Does informal caregiving lead to parental burnout? Comparing parents having (or not) children with mental and physical issues. Front Psychol.

[8] Kazak AE, Kassam-Adams N, Schneider S, Zelikovsky N, Alderfer MA, Rourke M (2006). An integrative model of pediatric medical traumatic stress. J Pediatr Psychol.

[9] Alsaigh R, Coyne I (2019). Mothers’ experiences of caring for children receiving growth hormone treatment. J Pediatr Nurs..

[10] Fitzgerald HE, Robinson LR, Cabrera N, Segal L (2021). Fathers and families: risk and resilience. An Introduction. Advers Resil Sci.

[11] Celano M, Bakeman R, Gaytan O, Smith CO, Koci A, Henderson S (2008). Caregiver depressive symptoms and observed family interaction in low-income children with persistent asthma. Fam Process.

[12] Eckshtain D, Ellis DA, Kolmodin K, Naar-King S (2010). The effects of parental depression and parenting practices on depressive symptoms and metabolic control in urban youth with insulin dependent diabetes. J Pediatr Psychol.

[13] Lord JH, Rumburg TM, Jaser SS (2015). Staying positive: positive affect as a predictor of resilience in adolescents with type 1 diabetes. J Pediatr Psychol.

[14] Yi-Frazier JP, Fladeboe K, Klein V, Eaton L, Wharton C, McCauley E (2017). Promoting resilience in stress management for parents (PRISM-P): an intervention for caregivers of youth with serious illness. Fam Syst Health.

[15] Okafor M, Sarpong DF, Ferguson A, Satcher D (2014). Improving health outcomes of children through effective parenting: model and methods. Int J Environ Res Public Health.

[16] Howard Sharp KM, Willard VW, Okado Y, Tillery R, Barnes S, Long A (2015). Profiles of connectedness: processes of resilience and growth in children with cancer. J Pediatr Psychol.

[17] Kalapurakkel SA, Carpino E, Lebel AE, Simons L (2015). “Pain can’t stop me”: Examining pain self-efficacy and acceptance as resilience processes among youth with chronic headache. J Pediatr Psychol..

[18] Douma M, Bouman CP, van Oers HA, Maurice-Stam H, Haverman L, Grootenhuis MA (2020). Matching psychosocial support needs of parents of a child with a chronic illness to a feasible intervention. Matern Child Health J..

[19] Haverkamp F, Gasteyger C (2011). A review of biopsychosocial strategies to prevent and overcome early-recognized poor adherence in growth hormone therapy of children. J Med Econ.

[20] Raina P, O’Donnell M, Schwellnus H, Rosenbaum P, King G, Brehaut J (2004). Caregiving process and caregiver burden: conceptual models to guide research and practice. BMC Pediatr..

[21] Gouveia MJ, Carona C, Canavarro MC, Moreira H (2016). Self-compassion and dispositional mindfulness are associated with parenting styles and parenting stress: the mediating role of mindful parenting. Mindfulness.

[22] Butler AC, Chapman JE, Forman EM, Beck AT (2006). The empirical status of cognitive-behavioral therapy: a review of meta-analyses. Clin Psychol Rev.

[23] Segal ZV, Teasdale JD, Williams JMG. Mindfulness-based cognitive therapy: theoretical rationale and empirical status. 2004;

[24] Townshend K, Jordan Z, Stephenson M, Tsey K (2016). The effectiveness of mindful parenting programs in promoting parents’ and children’s wellbeing: a systematic review. JBI Evid Synth.

[25] Sanders MR (2012). Development, evaluation, and multinational dissemination of the triple p-positive parenting program. Annu Rev Clin Psychol..

[26] Rosland A-M, Piette JD (2010). Emerging models for mobilizing family support for chronic disease management: a structured review. Chronic Illn.

[27] Berger AM, Mooney K, Alvarez-Perez A, Breitbart WS, Carpenter KM, Cella D, et al. Cancer-Related Fatigue, Version 2.2015. J Natl Compr Cancer Netw JNCCN. 2015 Aug;13(8):1012–39. 10.6004/jnccn.2015.0122 PMC5499710 26285247

[28] Mayberry LS, Berg CA, Harper KJ, Osborn CY. The design, usability, and feasibility of a family-focused diabetes self-care support mHealth intervention for diverse, low-income adults with type 2 diabetes. J Diabetes Res. 2016;2016. 10.1155/2016/7586385 PMC5116505 27891524

[29] Lerret SM, Schiffman R, White-Traut R, Medoff-Cooper B, Ahamed SI, Adib R, et al. Feasibility and Acceptability of a mHealth Self-Management Intervention for Pediatric Transplant Families. West J Nurs Res. 2021;01939459211024656. 10.1177/01939459211024656 PMC8688578 34154460

[30] Lerret SM, White-Traut R, Medoff-Cooper B, Simpson P, Adib R, Ahamed SI (2020). Pilot study protocol of a mHealth self-management intervention for family members of pediatric transplant recipients. Res Nurs Health.

[31] Canter KS, Christofferson J, Scialla MA, Kazak AE (2019). Technology-focused family interventions in pediatric chronic illness: a systematic review. J Clin Psychol Med Settings..

[32] Thabane L, Lancaster G. A guide to the reporting of protocols of pilot and feasibility trials. Springer; 2019. 10.1186/s40814-019-0423-8 PMC6393983 30858987

[33] Bedrov A, Bulaj G (2018). Improving self-esteem with motivational quotes: opportunities for digital health technologies for people with chronic disorders. Front Psychol.

[34] Koledova E, Le Masne Q, Spataru A, Bagha M, Dixon D (2021). Digital health in the management of pediatric growth hormone therapy - 10 years of developments. Stud Health Technol Inform.

[35] Dahlgren J (2008). Easypod™ a new electronic injection device for growth hormone. Expert Rev Med Dev.

[36] Sabben G, Mudhune V, Ondeng’e K, Odero I, Ndivo R, Akelo V, et al. A smartphone game to prevent HIV among young Africans (Tumaini): assessing intervention and study acceptability among adolescents and their parents in a randomized controlled trial. JMIR MHealth UHealth. 2019;7(5):e13049 10.2196/13049 PMC6547768 31115348

[37] Braun V, Clarke V (2006). Using thematic analysis in psychology. Qual Res Psychol.

[38] Bögels S, Restifo K. Mindful parenting: A guide for mental health practitioners. Springer; 2013.

[39] Hors-Fraile S, Benjumea FJN, Hernández LC, Ruiz FO, Fernandez-Luque L. Design of two combined health recommender systems for tailoring messages in a smoking cessation app. In: International Workshop on Engendering Health with RecSys co-located with ACM RecSys Boston, MA, USA. 2016.

[40] Arnao MDR, Sánchez AR, López ID, Fernández JR, de la Vega JAB, Fernández DY (2019). Adherence and long-term outcomes of growth hormone therapy with easypod™ in pediatric subjects: Spanish ECOS study. Endocr Connect.

[41] de Arriba MA, Muñiz VC, Saez JJA, Beisti A, Llovet E, Aizpún JIL (2021). Impact of adherence on growth response during the first 2 years of growth hormone treatment. Endocrine.

[42] Mohr DC, Schueller SM, Montague E, Burns MN, Rashidi P (2014). The Behavioral Intervention Technology Model: An Integrated Conceptual and Technological Framework for eHealth and mHealth Interventions. J Med Internet Res..

[43] Cedefop ETF. UNESCO, & UNESCO UIL.(2019). Global inventory of regional and national qualifications frameworks. UNESCO Inst Lifelong Learn LUI UNESCO Eur Train Found ETF Eur Cent Dev Vocat Train Cedefop. 2.

[44] Lovibond PF, Lovibond SH (1995). The structure of negative emotional states: comparison of the depression anxiety stress scales (DASS) with the beck depression and anxiety inventories. Behav Res Ther.

[45] Daza P, Novy DM, Stanley MA, Averill P (2002). The depression anxiety stress scale-21: Spanish translation and validation with a Hispanic sample. J Psychopathol Behav Assess.

[46] Bangor A, Kortum PT, Miller JT (2008). An empirical evaluation of the system usability scale. Int J Human-Computer Interact..

[47] López-Gómez I, Hervás G, Vázquez C (2015). Adaptación de la “Escala de afecto positivo y negativo”(PANAS) en una muestra general española. Psicol Conduct.

[48] Keyes CL, Wissing M, Potgieter JP, Temane M, Kruger A, Van Rooy S (2008). Evaluation of the mental health continuum–short form (MHC–SF) in setswana-speaking South Africans. Clin Psychol Psychother.

[49] Echeverría G, Torres M, Pedrals N, Padilla O, Rigotti A, Bitran M (2017). Validation of a Spanish version of the mental health continuum-short form questionnaire. Psicothema.

[50] Schwarzer R, Jerusalem M (1995). Generalized self-efficacy scale. Meas Health Psychol User’s Portf Causal Control Beliefs.

[51] Baessler J, Schwarzer R. Evaluación de la autoeficacia: Adaptación española de la escala de Autoeficacia General. Ansiedad Estrés. 1996;

[52] Bullinger M, Quitmann J, Power M, Herdman M, Mimoun E, DeBusk K (2013). Assessing the quality of life of health-referred children and adolescents with short stature: development and psychometric testing of the QoLISSY instrument. Health Qual Life Outcomes.

[53] Ravens-Sieberer U, Gosch A, Rajmil L, Erhart M, Bruil J, Duer W (2005). KIDSCREEN-52 quality-of-life measure for children and adolescents. Expert Rev Pharmacoecon Outcomes Res.

[54] Judd CM, Westfall J, Kenny DA (2017). Experiments with more than one random factor: Designs, analytic models, and statistical power. Annu Rev Psychol.

