# Supplementary material for: Digital health for emotional and self-management support of caregivers of children receiving growth hormone treatment: a feasibility study protocol
Source: BMC Med Inform Decis Mak. 2022 Aug 13;22:215. doi: 10.1186/s12911-022-01935-1 (PMC9375279; doi:10.1186/s12911-022-01935-1)
Supplement: Supplementary file 2 — Additional file 2. Semi-structured qualitative interview designed for the qualitative sub-study 1. [file 12911_2022_1935_MOESM2_ESM.pdf]

Semi-structured qualitative interview designed for the qualitative sub-study 1 (SS1)

Introductory part:

"First of all, we thank you very much the fact that you participate in this study. Your contribution is in all lights highly valuable. As mentioned before, in our first meeting, we acknowledge that being caregiver/parent of children with a growth condition may be stressful. Moreover, we also take into consideration that caregiving is an experience that may differ from person to person in great measure. For that reason, the goal of the current interview is to be able to understand in more detail you own experience as caregiver of a child with a growth condition and your opinion about how the app that you have used during the last month has or could help you. Said that, in the following, I will formulate several questions that I would like you to answer having in mind that there are no right or wrong responses. We are just interested in your opinions and thoughts. Of course, if you have any doubt or do not understand any question, do not hesitate to ask me at any moment."

Solution content related questions

- As a general impression, in which measure did you find the solution useful? (0-10)
- Which contents of the app have you found as more/less useful?
- What can you tell me about the information provided in the educational module?
- What do you think about the motivational recommenders?
- How did you find the emotional support information?
- How do you feel after engaging with the app? Which differences (if any) do you find as compared with before using it?

Solution content-format related questions

- In which measure do you find the solution easy or difficult to use?
- Have you experienced any problem when using the app? Could you provide some details?
- In your opinion, how understandable/clear is the information provided by the app?
- What about the design, how attractive do you find the app?
- Which features of the app did you (dis)like the most?
- If you could change or add anything to the solution, what would it be? Why?

Concluding remarks

- To which extent do you consider that your relationship with you child affects his/her adherence to the treatment? How would you say that affects it?
- In which measure do you think that supporting you as caregiver may have benefits on your child health? In this regard:
- What would you say are the advantages of this app?
- What do you miss in the app? How do you think that the app could be improved to help you to support your child adherence to the GHt?
